# 3,3′-Di-*tert*-butyl-1,1′-[1,3-phenyl­enebis(methyl­ene)]diurea

**DOI:** 10.1107/S1600536810005866

**Published:** 2010-02-20

**Authors:** Musabbir A. Saeed, Frank R. Fronczek, Md. Alamgir Hossain

**Affiliations:** a Department of Chemistry and Biochemistry, Jackson State University, Jackson, MS 39217, USA; b Department of Chemistry, Louisiana State University, Baton Rouge, LA 70803, USA

## Abstract

The title compound, C_18_H_30_N_4_O_2_, contains two *tert*-butyl urea groups, each connected to a benzene ring though a methyl­ene group. One of the groups occupies a position almost normal to the aromatic plane with a C—N—C—C torsion angle of −94.4 (4)°, while the other is considerably twisted from the ring with a C—N—C—C torsion angle of −136.1 (4)°. In the crystal, pairs of mol­ecules are connected to each other, forming centrosymmetric dimers in which two NH groups of one mol­ecule act as hydrogen-bond donors to one carbonyl O atom of the other mol­ecule. The dimers are linked into sheets parallel to (100) by N—H⋯O hydrogen bonds involving the remaining N—H and C=O groups.

## Related literature

For general background to urea-based compounds, see: Brooks *et al.* (2008 ▶); Carr *et al.* (1998 ▶); Chauhan *et al.* (2008 ▶); Gomez *et al.* (2005 ▶); Hiscock *et al.* (2009 ▶); Hossain (2008 ▶); Kyne *et al.* (2001 ▶); Lorenzo *et al.* (2009 ▶); Pérez-Casas & Yatsimirsky (2008 ▶); Tejeda *et al.* (2000 ▶); Ghosh *et al.* (2007 ▶). For related structures, see: Jose *et al.* (2007 ▶); Lo & Ng (2008 ▶).
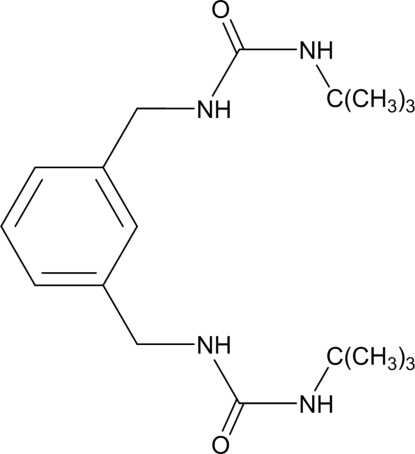


## Experimental

### 

#### Crystal data

C_18_H_30_N_4_O_2_
*M* *_r_* = 334.46
Orthorhombic, 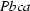

*a* = 18.070 (4) Å
*b* = 11.760 (3) Å
*c* = 18.221 (3) Å
*V* = 3872.0 (15) Å^3^
*Z* = 8
Mo *K*α radiation
μ = 0.08 mm^−1^
*T* = 90 K
0.20 × 0.10 × 0.07 mm

#### Data collection

Nonius KappaCCD diffractometer with an Oxford Cryosystems Cryostream cooler
43147 measured reflections
3781 independent reflections
2158 reflections with *I* > 2σ(*I*)
*R*_int_ = 0.081

#### Refinement

*R*[*F* ^2^ > 2σ(*F* ^2^)] = 0.081
*wR*(*F* ^2^) = 0.235
*S* = 1.03
3781 reflections
235 parameters
4 restraints
H atoms treated by a mixture of independent and constrained refinement
Δρ_max_ = 0.86 e Å^−3^
Δρ_min_ = −0.31 e Å^−3^

### 

Data collection: *COLLECT* (Nonius, 2000 ▶); cell refinement: *DENZO*/*SCALEPACK* (Otwinowski & Minor 1997 ▶); data reduction: *DENZO*/*SCALEPACK*; program(s) used to solve structure: *SIR97* (Altomare *et al.*, 1999 ▶); program(s) used to refine structure: *SHELXL97* (Sheldrick, 2008 ▶); molecular graphics: *ORTEP-3 for Windows* (Farrugia, 1997 ▶); software used to prepare material for publication: *SHELXL97*.

## Supplementary Material

Crystal structure: contains datablocks global, I. DOI: 10.1107/S1600536810005866/ci5033sup1.cif

Structure factors: contains datablocks I. DOI: 10.1107/S1600536810005866/ci5033Isup2.hkl

Additional supplementary materials:  crystallographic information; 3D view; checkCIF report

## Figures and Tables

**Table 1:** Hydrogen-bond geometry (Å, °)

*D*—H⋯*A*	*D*—H	H⋯*A*	*D*⋯*A*	*D*—H⋯*A*
N1—H1*N*⋯O2^i^	0.82 (2)	2.12 (2)	2.909 (4)	162 (4)
N2—H2*N*⋯O2^i^	0.81 (2)	2.28 (2)	3.034 (4)	153 (4)
N3—H3*N*⋯O1^ii^	0.86 (2)	2.15 (2)	2.941 (4)	154 (4)
N4—H4*N*⋯O1^ii^	0.81 (2)	2.12 (2)	2.889 (4)	160 (4)

Symmetry codes: (i) 
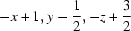
; (ii) 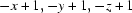.

**Fig. 1.**
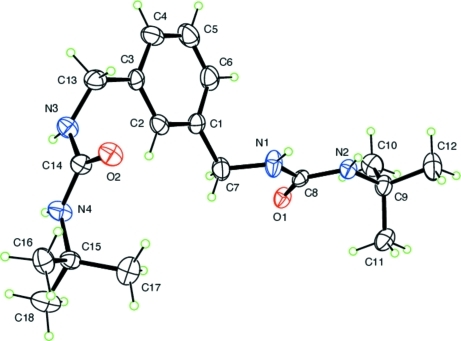
The molecular structure of the title compound. Displacement ellipsoids are drawn at the 50% probability level.

**Fig. 2.**
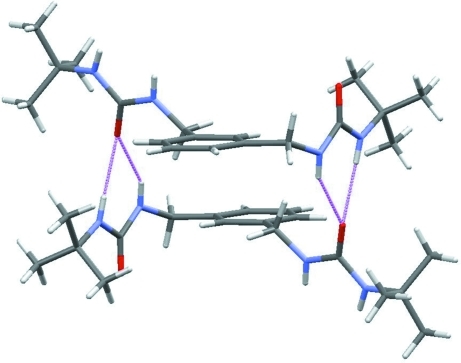
A view of the centrosymmetric dimer. Colour code: O, red; N, blue; C, deep gray; and H, light gray.

**Fig. 3.**
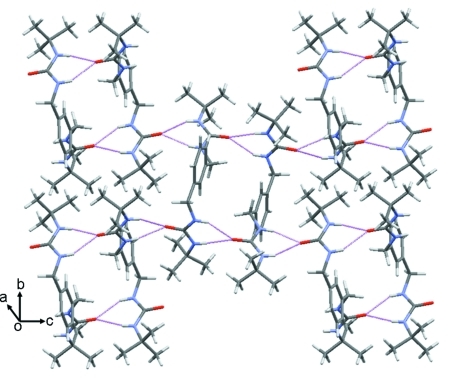
Packing diagram of the title compound viewed along a axis.

## supplementary crystallographic information

### Comment

Because of the ability of urea functional groups to form hydrogen bonds with an anion, urea-based compounds are known as effective hosts for a variety of anions (Brooks *et al.*, 2008; Carr *et al.*, 1998; Chauhan *et al.*, 2008; Hiscock *et al.*, 2009; Lorenzo *et al.*, 2009; Tejeda *et al.*, 2000) as well as neutral species (Kyne *et al.*, 2001) and are often used for colorimetric detection for a specific anion in solution (Ghosh *et al.*, 2007; Pérez-Casas *et al.*, 2008). For example, simple acyclic ligands with mono-functional urea or thiourea groups are known to form 1:1 complexes with carboxylates, halides and phosphate in DMSO (Gomez *et al.*, 2005). Encapsulation of sulfate anion was also structurally identified within the cavity formed by two tren-based urea ligands (Jose *et al.*, 2007). In an effort to design multifunctional anion receptors (Hossain, 2008), we synthesized a urea-based compound containing two urea units, that can be useful in anion binding.

Single crystal X-ray analysis reveals that the bis-urea cleft crystallized in an orthorhombic space group without the involvement of solvent molecules. As illustrated in Fig. 1, the carbonyl groups of the two urea fragments are oriented in opposite directions. The methylene groups connected to the aromatic units are almost co-planar with the NH groups, as indicated by C13—N3—C14—N4 and C7—N1—C8—N2 torsion angles of -164.8 (3) and 177.6 (3)°, respectively. The NH groups are oriented almost perpendicular to the aromatic plane.

There are no intramolecular hydrogen bonding in the molecule, however, each C═O group forms two hydrogen bonds with two adjacent NH fragments resulting in the formation of centrosymmetric dimers with N···O distances of 2.889 (4) Å and 2.941 (4) Å (Fig. 2 and Table 1). Each dimer is then connected with four adjacent dimers forming a sheet parallel to the (100) (Fig. 3). Similar intermolecular bonding was previously reported for a mono-functional urea-based compound (Lo & Ng, 2008).

### Experimental

To a solution of *m*-xylylenediamine (0.10 g, 1.0 mmol) in CH_3_CN (20 ml) was added two equivalents of *tert*-butyl isocyanate (0.20 g, 2.0 mmol) and the mixture was stirred overnight at room temperature. The white precipitate formed was separated by filtration, washed by diethyl ether, and dried under vacuum. Yield 40%. ^1^H NMR (500 MHz, CDCl_3_, TMS): δ 7.25-7.12 (ArH, m, 4H), 4.84(CH_2_NH, t, J = 5 Hz, 2H), 4.64 (CNH, s, 2H), 4.21 (ArCH_2_, d, J = 5 Hz, 4H), 1.30 (CCH_3_, s, 18H). A small portion of the sample was re-dissolved in CHCl_3_, and crystals suitable for X-ray analysis were grown by slow evaporation of the solvent at room temperature.

### Refinement

N-bound H atoms were located in a difference map and their coordinates were refined with a N–H distance restraint of 0.81 (1) Å. C-bound H atoms were placed in idealized positions [C–H = 0.95–0.99 Å] and thereafter treated as riding. *U*_iso_(H) values were assigned as 1.2 times *U*_eq_ of the attached atom (1.5 for methyl). A torsional parameter was refined for each methyl group. The highest residual peak is located 1.50 Å from O2.

### Crystal data

**Table:**

C_18_H_30_N_4_O_2_	*F*(000) = 1456
*M**_r_* = 334.46	*D*_x_ = 1.147 Mg m^−^^3^
Orthorhombic, *P**b**c**a*	Mo *K*α radiation, λ = 0.71073 Å
Hall symbol: -P 2ac 2ab	Cell parameters from 4187 reflections
*a* = 18.070 (4) Å	θ = 2.5–26.0°
*b* = 11.760 (3) Å	µ = 0.08 mm^−^^1^
*c* = 18.221 (3) Å	*T* = 90 K
*V* = 3872.0 (15) Å^3^	Plate, colourless
*Z* = 8	0.20 × 0.10 × 0.07 mm

### Data collection

**Table:**

Nonius KappaCCD diffractometer with an Oxford Cryosystems Cryostream cooler	2158 reflections with *I* > 2σ(*I*)
Radiation source: fine-focus sealed tube	*R*_int_ = 0.081
graphite	θ_max_ = 26.0°, θ_min_ = 2.8°
ω and φ scans	*h* = −22→22
43147 measured reflections	*k* = −14→14
3781 independent reflections	*l* = −22→22

### Refinement

**Table:**

Refinement on *F*^2^	Primary atom site location: structure-invariant direct methods
Least-squares matrix: full	Secondary atom site location: difference Fourier map
*R*[*F*^2^ > 2σ(*F*^2^)] = 0.081	Hydrogen site location: inferred from neighbouring sites
*wR*(*F*^2^) = 0.235	H atoms treated by a mixture of independent and constrained refinement
*S* = 1.03	*w* = 1/[σ^2^(*F*_o_^2^) + (0.1146*P*)^2^ + 3.4665*P*] where *P* = (*F*_o_^2^ + 2*F*_c_^2^)/3
3781 reflections	(Δ/σ)_max_ = 0.001
235 parameters	Δρ_max_ = 0.86 e Å^−^^3^
4 restraints	Δρ_min_ = −0.31 e Å^−^^3^

### Special details

**Table:**

Geometry. All esds (except the esd in the dihedral angle between two l.s. planes) are estimated using the full covariance matrix. The cell esds are taken into account individually in the estimation of esds in distances, angles and torsion angles; correlations between esds in cell parameters are only used when they are defined by crystal symmetry. An approximate (isotropic) treatment of cell esds is used for estimating esds involving l.s. planes.
Refinement. Refinement of *F*^2^ against ALL reflections. The weighted *R*-factor wR and goodness of fit *S* are based on *F*^2^, conventional *R*-factors *R* are based on *F*, with *F* set to zero for negative *F*^2^. The threshold expression of *F*^2^ > σ(*F*^2^) is used only for calculating *R*-factors(gt) etc. and is not relevant to the choice of reflections for refinement. *R*-factors based on *F*^2^ are statistically about twice as large as those based on *F*, and *R*- factors based on ALL data will be even larger.

### Fractional atomic coordinates and isotropic or equivalent isotropic displacement parameters (Å^2^)

**Table:**

	*x*	*y*	*z*	*U*_iso_*/*U*_eq_	
O1	0.34787 (13)	0.2316 (2)	0.50086 (12)	0.0278 (6)	
O2	0.60321 (15)	0.6965 (2)	0.74637 (13)	0.0341 (7)	
N1	0.41055 (18)	0.2793 (3)	0.60384 (16)	0.0374 (9)	
H1N	0.414 (2)	0.267 (4)	0.6479 (11)	0.045*	
N2	0.32387 (16)	0.1387 (3)	0.60835 (15)	0.0252 (7)	
H2N	0.333 (2)	0.138 (3)	0.6520 (11)	0.030*	
N3	0.66516 (18)	0.6521 (3)	0.64143 (16)	0.0302 (8)	
H3N	0.664 (2)	0.664 (3)	0.5948 (11)	0.036*	
N4	0.58391 (18)	0.8006 (3)	0.64105 (15)	0.0306 (8)	
H4N	0.593 (2)	0.798 (3)	0.5976 (11)	0.037*	
C1	0.5353 (2)	0.3564 (3)	0.59313 (18)	0.0265 (8)	
C2	0.5746 (2)	0.4512 (3)	0.61638 (18)	0.0284 (9)	
H2	0.5515	0.5237	0.6160	0.034*	
C3	0.6492 (2)	0.4409 (3)	0.64085 (18)	0.0260 (8)	
C4	0.6807 (2)	0.3341 (3)	0.6401 (2)	0.0359 (10)	
H4	0.7304	0.3254	0.6562	0.043*	
C5	0.6429 (3)	0.2405 (4)	0.6169 (2)	0.0472 (12)	
H5	0.6661	0.1680	0.6165	0.057*	
C6	0.5719 (2)	0.2520 (4)	0.5945 (2)	0.0399 (10)	
H6	0.5459	0.1860	0.5790	0.048*	
C7	0.4566 (2)	0.3648 (3)	0.5684 (2)	0.0362 (10)	
H7A	0.4372	0.4414	0.5801	0.043*	
H7B	0.4542	0.3545	0.5146	0.043*	
C8	0.35958 (18)	0.2172 (3)	0.56722 (17)	0.0221 (8)	
C9	0.2612 (2)	0.0693 (3)	0.58226 (18)	0.0303 (9)	
C10	0.2850 (2)	−0.0051 (4)	0.5169 (2)	0.0393 (10)	
H10A	0.2983	0.0437	0.4753	0.059*	
H10B	0.2440	−0.0551	0.5027	0.059*	
H10C	0.3278	−0.0513	0.5310	0.059*	
C11	0.1961 (2)	0.1455 (4)	0.5611 (2)	0.0376 (10)	
H11A	0.1792	0.1876	0.6044	0.056*	
H11B	0.1556	0.0986	0.5423	0.056*	
H11C	0.2119	0.1993	0.5231	0.056*	
C12	0.2391 (3)	−0.0088 (4)	0.6458 (2)	0.0457 (12)	
H12A	0.2818	−0.0548	0.6606	0.069*	
H12B	0.1988	−0.0590	0.6300	0.069*	
H12C	0.2225	0.0374	0.6875	0.069*	
C13	0.6909 (2)	0.5427 (3)	0.6683 (2)	0.0333 (10)	
H13A	0.6883	0.5433	0.7225	0.040*	
H13B	0.7435	0.5339	0.6545	0.040*	
C14	0.6158 (2)	0.7160 (3)	0.68049 (18)	0.0272 (8)	
C15	0.5256 (2)	0.8778 (3)	0.66675 (18)	0.0259 (8)	
C16	0.5559 (2)	0.9634 (3)	0.7207 (2)	0.0362 (10)	
H16A	0.5762	0.9234	0.7634	0.054*	
H16B	0.5161	1.0143	0.7366	0.054*	
H16C	0.5951	1.0079	0.6972	0.054*	
C17	0.4608 (2)	0.8133 (4)	0.6996 (2)	0.0417 (11)	
H17A	0.4416	0.7590	0.6635	0.063*	
H17B	0.4216	0.8671	0.7131	0.063*	
H17C	0.4773	0.7723	0.7435	0.063*	
C18	0.4988 (3)	0.9395 (4)	0.5971 (2)	0.0434 (11)	
H18A	0.5408	0.9771	0.5733	0.065*	
H18B	0.4616	0.9966	0.6105	0.065*	
H18C	0.4768	0.8843	0.5632	0.065*	

### Atomic displacement parameters (Å^2^)

**Table:**

	*U*^11^	*U*^22^	*U*^33^	*U*^12^	*U*^13^	*U*^23^
O1	0.0263 (14)	0.0416 (16)	0.0156 (13)	−0.0042 (12)	0.0019 (10)	0.0011 (10)
O2	0.0435 (17)	0.0359 (16)	0.0228 (14)	0.0026 (13)	−0.0013 (11)	0.0004 (11)
N1	0.038 (2)	0.054 (2)	0.0201 (16)	−0.0192 (18)	−0.0036 (14)	0.0074 (15)
N2	0.0255 (17)	0.0353 (18)	0.0147 (14)	−0.0036 (14)	−0.0021 (12)	0.0024 (13)
N3	0.0365 (19)	0.0284 (18)	0.0256 (16)	0.0014 (16)	0.0007 (14)	0.0007 (13)
N4	0.042 (2)	0.0358 (19)	0.0139 (14)	0.0110 (16)	0.0045 (14)	0.0045 (13)
C1	0.028 (2)	0.030 (2)	0.0214 (18)	−0.0013 (18)	0.0065 (15)	−0.0001 (15)
C2	0.035 (2)	0.028 (2)	0.0223 (18)	0.0022 (18)	0.0057 (16)	0.0035 (15)
C3	0.028 (2)	0.028 (2)	0.0221 (18)	−0.0024 (17)	0.0082 (15)	0.0018 (15)
C4	0.039 (2)	0.034 (2)	0.034 (2)	0.011 (2)	0.0043 (18)	0.0051 (17)
C5	0.054 (3)	0.028 (2)	0.059 (3)	0.009 (2)	0.020 (2)	0.008 (2)
C6	0.044 (3)	0.034 (2)	0.042 (2)	−0.001 (2)	0.018 (2)	0.0023 (19)
C7	0.034 (2)	0.039 (2)	0.036 (2)	−0.012 (2)	−0.0053 (18)	0.0105 (18)
C8	0.0182 (18)	0.030 (2)	0.0179 (18)	0.0015 (16)	0.0030 (14)	−0.0008 (14)
C9	0.028 (2)	0.037 (2)	0.0263 (19)	−0.0075 (18)	−0.0020 (16)	0.0036 (16)
C10	0.043 (3)	0.034 (2)	0.040 (2)	−0.005 (2)	−0.0047 (19)	−0.0060 (19)
C11	0.026 (2)	0.050 (3)	0.037 (2)	−0.005 (2)	−0.0007 (17)	−0.0004 (19)
C12	0.046 (3)	0.054 (3)	0.037 (2)	−0.021 (2)	−0.004 (2)	0.010 (2)
C13	0.032 (2)	0.037 (2)	0.030 (2)	0.0033 (19)	−0.0038 (17)	0.0035 (17)
C14	0.031 (2)	0.028 (2)	0.0233 (19)	0.0032 (18)	0.0027 (16)	−0.0022 (16)
C15	0.029 (2)	0.026 (2)	0.0231 (18)	0.0052 (17)	0.0016 (15)	0.0005 (15)
C16	0.046 (3)	0.031 (2)	0.032 (2)	−0.004 (2)	0.0063 (18)	−0.0001 (16)
C17	0.040 (3)	0.047 (3)	0.038 (2)	−0.008 (2)	0.0044 (19)	−0.0069 (19)
C18	0.055 (3)	0.043 (3)	0.033 (2)	0.018 (2)	−0.002 (2)	0.0009 (18)

### Geometric parameters (Å, °)

**Table:**

O1—C8	1.239 (4)	C7—H7B	0.99
O2—C14	1.243 (4)	C9—C11	1.527 (5)
N1—C8	1.352 (5)	C9—C12	1.530 (5)
N1—C7	1.455 (5)	C9—C10	1.539 (5)
N1—H1N	0.819 (18)	C10—H10A	0.98
N2—C8	1.353 (4)	C10—H10B	0.98
N2—C9	1.475 (5)	C10—H10C	0.98
N2—H2N	0.813 (18)	C11—H11A	0.98
N3—C14	1.367 (5)	C11—H11B	0.98
N3—C13	1.452 (5)	C11—H11C	0.98
N3—H3N	0.860 (18)	C12—H12A	0.98
N4—C14	1.355 (5)	C12—H12B	0.98
N4—C15	1.468 (5)	C12—H12C	0.98
N4—H4N	0.809 (18)	C13—H13A	0.99
C1—C2	1.388 (5)	C13—H13B	0.99
C1—C6	1.395 (5)	C15—C16	1.510 (5)
C1—C7	1.495 (5)	C15—C17	1.518 (5)
C2—C3	1.426 (5)	C15—C18	1.540 (5)
C2—H2	0.95	C16—H16A	0.98
C3—C4	1.380 (5)	C16—H16B	0.98
C3—C13	1.499 (5)	C16—H16C	0.98
C4—C5	1.362 (6)	C17—H17A	0.98
C4—H4	0.95	C17—H17B	0.98
C5—C6	1.352 (6)	C17—H17C	0.98
C5—H5	0.95	C18—H18A	0.98
C6—H6	0.95	C18—H18B	0.98
C7—H7A	0.99	C18—H18C	0.98
			
C8—N1—C7	122.9 (3)	C9—C10—H10C	109.5
C8—N1—H1N	117 (3)	H10A—C10—H10C	109.5
C7—N1—H1N	120 (3)	H10B—C10—H10C	109.5
C8—N2—C9	124.4 (3)	C9—C11—H11A	109.5
C8—N2—H2N	117 (3)	C9—C11—H11B	109.5
C9—N2—H2N	118 (3)	H11A—C11—H11B	109.5
C14—N3—C13	121.4 (3)	C9—C11—H11C	109.5
C14—N3—H3N	114 (3)	H11A—C11—H11C	109.5
C13—N3—H3N	119 (3)	H11B—C11—H11C	109.5
C14—N4—C15	126.2 (3)	C9—C12—H12A	109.5
C14—N4—H4N	114 (3)	C9—C12—H12B	109.5
C15—N4—H4N	119 (3)	H12A—C12—H12B	109.5
C2—C1—C6	117.3 (4)	C9—C12—H12C	109.5
C2—C1—C7	121.7 (3)	H12A—C12—H12C	109.5
C6—C1—C7	121.0 (4)	H12B—C12—H12C	109.5
C1—C2—C3	120.8 (3)	N3—C13—C3	115.7 (3)
C1—C2—H2	119.6	N3—C13—H13A	108.3
C3—C2—H2	119.6	C3—C13—H13A	108.3
C4—C3—C2	117.7 (4)	N3—C13—H13B	108.3
C4—C3—C13	121.5 (4)	C3—C13—H13B	108.3
C2—C3—C13	120.8 (3)	H13A—C13—H13B	107.4
C5—C4—C3	122.1 (4)	O2—C14—N4	124.7 (3)
C5—C4—H4	119.0	O2—C14—N3	121.4 (3)
C3—C4—H4	119.0	N4—C14—N3	113.9 (3)
C6—C5—C4	119.3 (4)	N4—C15—C16	111.0 (3)
C6—C5—H5	120.3	N4—C15—C17	111.8 (3)
C4—C5—H5	120.3	C16—C15—C17	110.9 (3)
C5—C6—C1	122.9 (4)	N4—C15—C18	104.7 (3)
C5—C6—H6	118.6	C16—C15—C18	109.7 (3)
C1—C6—H6	118.6	C17—C15—C18	108.6 (3)
N1—C7—C1	111.4 (3)	C15—C16—H16A	109.5
N1—C7—H7A	109.3	C15—C16—H16B	109.5
C1—C7—H7A	109.3	H16A—C16—H16B	109.5
N1—C7—H7B	109.3	C15—C16—H16C	109.5
C1—C7—H7B	109.3	H16A—C16—H16C	109.5
H7A—C7—H7B	108.0	H16B—C16—H16C	109.5
O1—C8—N1	121.6 (3)	C15—C17—H17A	109.5
O1—C8—N2	123.5 (3)	C15—C17—H17B	109.5
N1—C8—N2	114.8 (3)	H17A—C17—H17B	109.5
N2—C9—C11	110.3 (3)	C15—C17—H17C	109.5
N2—C9—C12	106.8 (3)	H17A—C17—H17C	109.5
C11—C9—C12	110.0 (3)	H17B—C17—H17C	109.5
N2—C9—C10	110.4 (3)	C15—C18—H18A	109.5
C11—C9—C10	110.7 (3)	C15—C18—H18B	109.5
C12—C9—C10	108.5 (3)	H18A—C18—H18B	109.5
C9—C10—H10A	109.5	C15—C18—H18C	109.5
C9—C10—H10B	109.5	H18A—C18—H18C	109.5
H10A—C10—H10B	109.5	H18B—C18—H18C	109.5
			
C6—C1—C2—C3	−0.3 (5)	C9—N2—C8—O1	−7.1 (5)
C7—C1—C2—C3	179.0 (3)	C9—N2—C8—N1	173.7 (3)
C1—C2—C3—C4	0.4 (5)	C8—N2—C9—C11	−61.4 (4)
C1—C2—C3—C13	−178.0 (3)	C8—N2—C9—C12	179.0 (3)
C2—C3—C4—C5	0.1 (5)	C8—N2—C9—C10	61.2 (4)
C13—C3—C4—C5	178.4 (4)	C14—N3—C13—C3	94.4 (4)
C3—C4—C5—C6	−0.6 (6)	C4—C3—C13—N3	157.3 (3)
C4—C5—C6—C1	0.7 (6)	C2—C3—C13—N3	−24.4 (5)
C2—C1—C6—C5	−0.3 (5)	C15—N4—C14—O2	−4.9 (6)
C7—C1—C6—C5	−179.5 (4)	C15—N4—C14—N3	175.7 (3)
C8—N1—C7—C1	−136.1 (4)	C13—N3—C14—O2	15.8 (6)
C2—C1—C7—N1	−131.0 (4)	C13—N3—C14—N4	−164.8 (3)
C6—C1—C7—N1	48.2 (5)	C14—N4—C15—C16	72.8 (5)
C7—N1—C8—O1	−1.7 (6)	C14—N4—C15—C17	−51.5 (5)
C7—N1—C8—N2	177.6 (3)	C14—N4—C15—C18	−168.9 (4)

### Hydrogen-bond geometry (Å, °)

**Table:**

*D*—H···*A*	*D*—H	H···*A*	*D*···*A*	*D*—H···*A*
N1—H1N···O2^i^	0.82 (2)	2.12 (2)	2.909 (4)	162 (4)
N2—H2N···O2^i^	0.81 (2)	2.28 (2)	3.034 (4)	153 (4)
N3—H3N···O1^ii^	0.86 (2)	2.15 (2)	2.941 (4)	154 (4)
N4—H4N···O1^ii^	0.81 (2)	2.12 (2)	2.889 (4)	160 (4)

Symmetry codes: (i) −*x*+1, *y*−1/2, −*z*+3/2; (ii) −*x*+1, −*y*+1, −*z*+1.
